# Rapid cell-free CD4 enumeration using whole saliva

**DOI:** 10.1186/1742-4690-9-S1-P58

**Published:** 2012-05-25

**Authors:** Cynthia L Bristow, Mariya A Babayeva, Rozbeh Modarresi, Carole P McArthur, Santosh Kumar, Charles Awasom, Leo Ayuk, Annette Nhinda, Paul Achu, Ronald Winston

**Affiliations:** 1 Weill Cornell Medical College, New York, USA

## Introduction

The determination of CD4 counts in patients with HIV/AIDS is of paramount importance clinically to determine when to initiate anti-retroviral therapy (ART). ART slows disease progression, reduces viral load and significantly reduces HIV transmission. Currently, the only methods for obtaining CD4 counts use flow cytometry technology. Unfortunately, the latter technologies require fresh cells, reliable and stable electricity, a high degree of technical training for laboratory personnel, expensive instrumentation, and calibrated pipetting which is often unavailable in rural areas or in resource-limited environments. A rapid, inexpensive, point-of-care method would overcome a critical barrier to HIV prevention because it would have a major impact on the cost and health outcomes of patients as well as the frequency of HIV transmission, all of which markedly impact global economy. A commonly used cost-saving and time-saving laboratory strategy is to calculate, rather than measure certain blood values. For example, LDL levels are calculated using measured levels of total cholesterol, HDL, and triglycerides. Thus, identification of cell-free correlates that directly regulate the number of CD4^+^ T cells could provide an accurate method for calculating CD4 counts due to the physiological relevance of the correlates. We have recently determined that α_1_proteinase inhibitor (α_1_PI, α_1_antitrypsin) participates in regulating the number of CD4^+^ T cells in blood.

## Materials and methods

Stimulated saliva was collected from 20 female and 11 male HIV-1 subjects attending clinic for routine care in Cameroon. The α_1_PI Index was calculated as the ratio of α_1_PI activity versus protein content in saliva and compared to CD4 counts determined by the standard method, flow cytometry.

## Results

The α_1_PI Index in saliva correlated with CD4 counts determined by flow cytometry (r²=0.91, p<0.0001. n=31). An algorithm was developed (the α-test) based on the α_1_PI Index. The precision of the α-test was approximately 26 CD4 cells/ml, and the accuracy of the α-test was approximately 95%.

## Conclusions

The α-test is physiologically relevant to CD4 counts and can be performed using saliva thereby providing a noninvasive, accurate and precise point-of-care method for monitoring CD4 counts in endemic regions with no instrumentation at a cost-per-test that is less than a dollar.

